# Microwave Bandpass Filter Based on Mie-Resonance Extraordinary Transmission

**DOI:** 10.1371/journal.pone.0166696

**Published:** 2016-12-19

**Authors:** Xiaolong Pan, Haiyan Wang, Dezhao Zhang, Shuang Xun, Mengzhu Ouyang, Wentao Fan, Yunsheng Guo, Ye Wu, Shanguo Huang, Ke Bi, Ming Lei

**Affiliations:** 1 State Key Laboratory of Information Photonics and Optical Communications & School of Science, Beijing University of Posts and Telecommunications, Beijing, China; 2 School of Information and Communication Engineering, Beijing University of Posts and Telecommunications, Beijing, China; 3 Department of Physics, Inner Mongolia University of Science and Technology, Baotou, China; Northwestern Polytechnical University, CHINA

## Abstract

Microwave bandpass filter structure has been designed and fabricated by filling the periodically metallic apertures with dielectric particles. The microwave cannot transmit through the metallic subwavelength apertures. By filling the metallic apertures with dielectric particles, a transmission passband with insertion loss 2 dB appears at the frequency of 10–12 GHz. Both simulated and experimental results show that the passband is induced by the Mie resonance of the dielectric particles. In addition, the passband frequency can be tuned by the size and the permittivity of the dielectric particles. This approach is suitable to fabricate the microwave bandpass filters.

## Introduction

Bandpass filters play essential roles in detecting and controlling the spectra of radio frequency signals in communications and radar systems[1]. For instance, these filters can be installed in the scanner receiver of electronic support measures systems to detect and classify incident signals for electronic countermeasures systems[2]. In recent years, many research on metamaterials has been done and new characteristics have been discovered, which provides a new way to design filters whose effective components, both active and passive, are difficult to realize by traditional approaches[3]. The filters based on metamaterials have been realized at the optical, terahertz (THz), and microwave bands by a multi-layer metamaterial structure[4], microstrip lines by complementary split ring resonators (CSRRs) etched on the ground plane[5–7], periodic 3-D (three-dimensional) metamaterial structure[8], or spoof surface plasmon polaritons based notch structure[9]. Relying on resonance of resonance-type metamaterials to influence the incoming electromagnetic waves, the aforementioned filters are realized or improved. However, the production process of the most filters is complex, which limits the potential application.

Recently, extraordinary optical transmission through a subwavelength aperture drilled in a thin metallic plate has inspired great theoretical and experimental interests for its potential applications in the fields of flat optics, nanolithography, chemical sensors, spectral filters, and optical trapping[10–12]. According to Bethe’s aperture theory, the transmission through a single aperture fairly decreases, incident electromagnetic waves cannot transmit through the plate[13–15]. However, by placing resonant-type metamaterial in the vicinity of the aperture or filling the aperture with high-permittivity material, the enhanced transmission through a subwavelength aperture can be achieved at a certain frequency, which opens a new way to design filters[16, 17]. Here, we report a microwave bandpass filter structure based on Mie-resonance extraordinary transmission. Without the dielectric particles, the incoming microwave cannot transmit through the metallic plate drilled subwavelength apertures. By filling the apertures with high-permittivity and low-loss dielectric particles, a microwave bandpass filter is achieved. A passband appears in the transmission spectra at 10–12 GHz and the insertion loss is 2 dB. The passband of the filter can be influenced by the size and permittivity of the dielectric particles, which provides an approach to fabricate filters.

## Experimental

**Fig 1:**
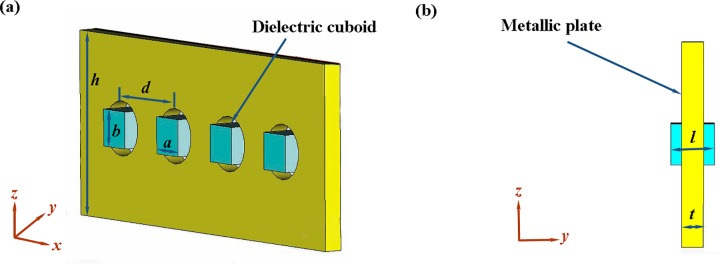
Schematic diagram of the microwave bandpass filter structure based on periodically metallic apertures filled with dielectric particles. (A) perspective view and (B) cross-section (*yz*-plane)

The dielectric material chosen for this work are Barium Strontium Titanate (BST) ceramic with dielectric loss of 0.002. Their relative permittivity *ε* is 120. Fig 1A and 1B shows the schematic diagram of the microwave bandpass filter structure composed of metallic plate and dielectric particles. The dimension of the chosen metallic plate is 5 × 1 × 10 mm^3^ (*d* × *t* × *h*). Apertures with a radius of *r* = 1.5 mm are drilled in the metallic plate. The transverse dimension of each dielectric particle designed in experiments is 2.0 × 2.0 mm^2^ (*a* × *b*). The length *l* of the dielectric particle is 1.8 mm, 1.9 mm and 2.0 mm, respectively. To measure the electromagnetic property of the microwave filter structure, the filter was placed in an X-band rectangular waveguide (22.86 × 10.16 mm^2^) with microwave incoming along the *y* axis, and the electric field and magnetic field along the *z* and *x* axes, respectively. The size of the unit cell for the filter structure is 5 × 5 × 10 mm^3^. Numerical predictions of the transmission spectra for this structure were calculated using the commercial time-domain package CST Microwave Studio TM. All the simulated setups were the same as those in the experiments. A vector network analyzer (N5230C, Agilent Technologies, USA) is chosen as the microwave measurement system for this work.

## Results and Discussion

By filling the subwavelength hole with a homogeneous material, the extraordinary transmission can be achieved. The electric field inside the hole full of homogeneous material can be expressed by[18]

$$E\left(r\right)=E^{\text{ext}}+ik\int_{S}ds[1+\frac{1}{k^{2}\varepsilon \mu }\nabla \nabla ]G\left(r-s\right)h\left(s\right)$$ (1)

$$G\left(r\right)=\frac{e^{ik\sqrt{\varepsilon \mu }r}}{r}$$ (2)

where *k = ω/c* is the momentum of light in vacuum, *S* represents the boundary of the homogeneous material, *E*^*ext*^ is external electric field, *ɛ* and *μ* are the permittivity and permeability of the homogeneous material, respectively. By interacting with the incident electromagnetic waves, the Mie resonance takes place in a dielectric particle. For a dielectric particle of a specified dimension and the permittivity, the Mie resonance dips appear in the transmission spectrum[19]. At the Mie resonance, the dielectric particle performs a magnetic dipole, with a field that is mainly localized within the particle[19, 20]. Therefore, a passband related to the Mie resonance will be obtained in the transmission of the proposed structure. For a dielectric cube, the lowest resonance frequency is given approximately by[21, 22]

$$f\propto \frac{1}{l}$$ (3)

where *ε*_*r*_ is the relative permittivity of the dielectric cube, *l* is the side length of the cube. Based on Eqs (1)–(3), it is obvious that the electric field energy depends strongly on the permittivity and the size of the located homogeneous material. The resonance frequency decreases as the *l* increases.

**Fig 2:**
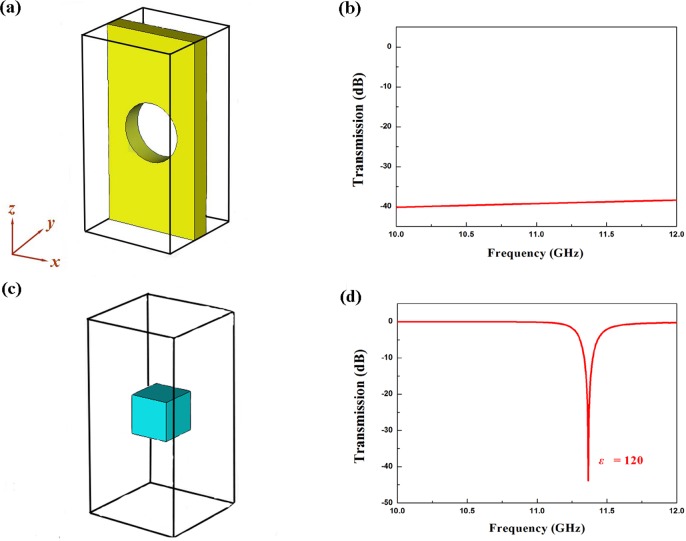
Schematic diagram and the simulated transmission spectrum. (A) Schematic diagram and (B) simulated transmission spectrum of metallic aperture unit cell. (C) Schematic diagram and (D) simulated transmission spectrum of single dielectric particle.

The schematic diagram for unit cell of metallic aperture without dielectric particle is shown in Fig 2A. The dimension of the unit cell is 5 × 5 × 10 mm^3^. As is shown in Fig 2B (Figure A in S1 File), the values of transmission coefficient is very small (about -42dB), which indicates the incident microwave is reflected by the metallic aperture unit cell and microwave cannot transmit through the metallic aperture. Fig 2C shows the schematic diagram of the single dielectric particle. Fig 2D shows the simulated transmission (Figure B in S1 File) spectrum of single dielectric particle. It can be seen that a transmission dip appears in 10–12 GHz, which is induced by Mie resonance[21].

**Fig 3:**
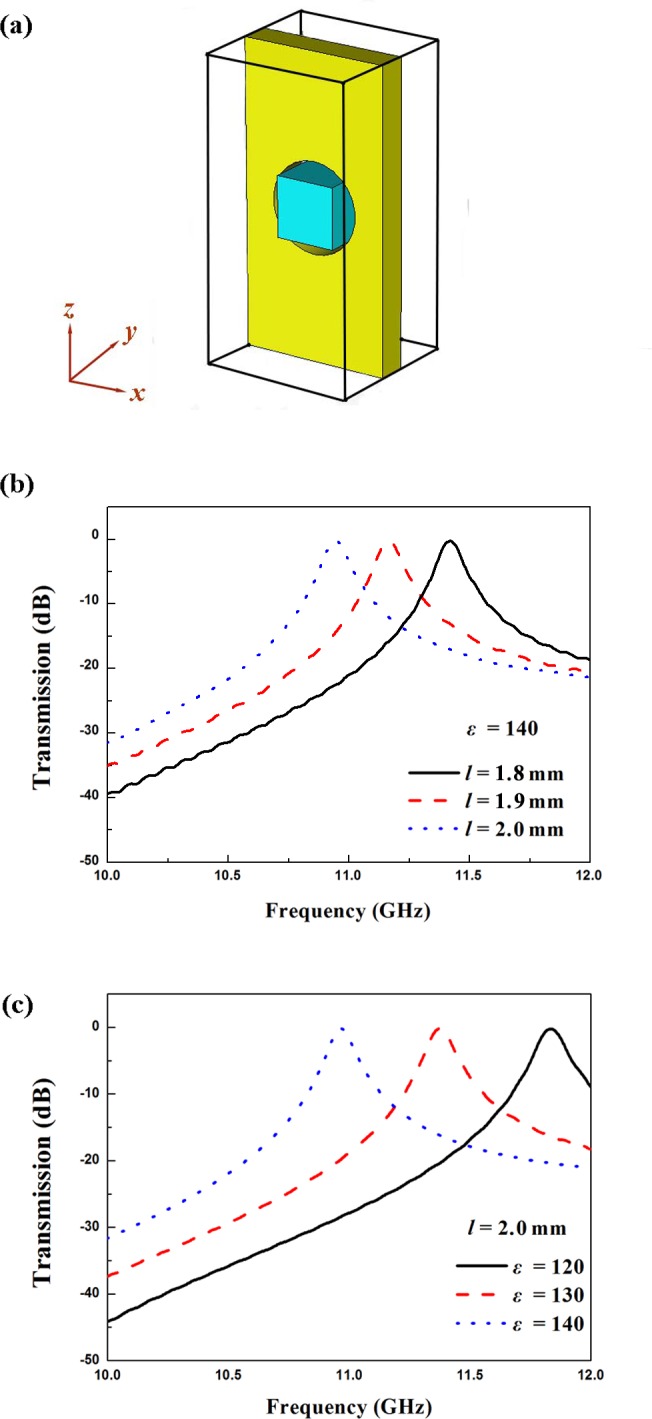
Schematic diagram and simulated transmission spectra for the unit cell of microwave filter structure. (A) Schematic diagram of the unit cell. (B) Simulated transmission spectra with a series of length *l*. (C) Simulated transmission spectra with a series of permittivity *ε*.

The schematic diagram for unit cell of metallic aperture filled with dielectric particle is shown in Fig 3A. Fig 3B (Figure C in S1 File) shows the simulated transmission spectra for unit cell with a series of dielectric particle length *l*. The permittivity *ε* is set as 140. For all cases, an obvious transmission passband induced by the Mie resonance of the dielectric particle appears. The insertion loss for the passband is about 0 dB, which indicates the unit cell can realized total transmission at a certain frequency. Meanwhile, as the length *l* of the dielectric particle increases from 1.8 mm to 2.0 mm, the center frequency of the passband decreases, which demonstrates the size of the dielectric particle can influence the transmission property of the unit cell. By placing dielectric particles with a series of permittivity *ε* into the aperture, the transmission spectra of the unit cell are simulated and shown in Fig 3C(Figure D in S1 File). The particle length *l* is set as 2.0 mm. It can be seen that, as the permittivity *ε* increases from 120 to 140, the center frequency of the passband decreases. The results shown in Fig 3 are in good agreement with those predicted by Eqs (1)–(3), which demonstrates this proposed structure is suitable for the application in microwave passband filters.

**Fig 4:**
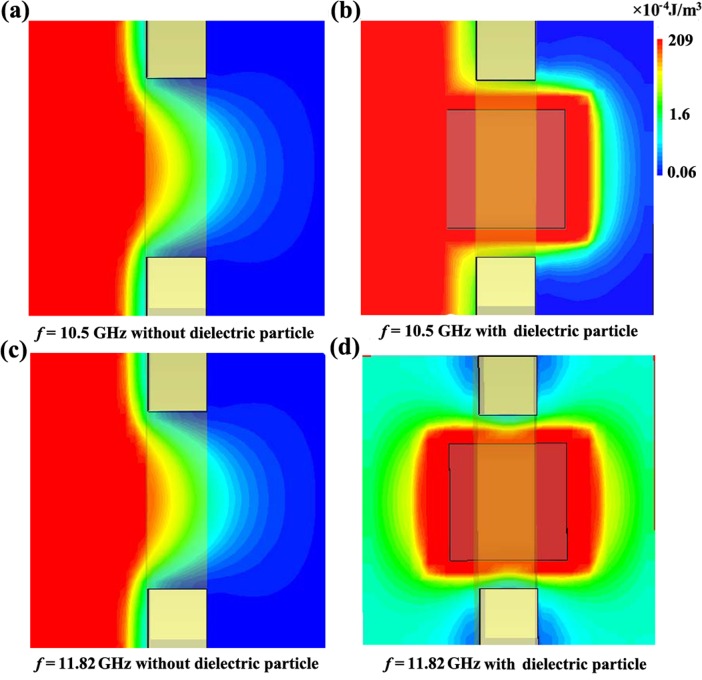
Simulated electric energy density distribution in the *xy*-plane. (A) Simulated electric energy density distribution in the *xy*-plane for metallic aperture unit cell at 10.5 GHz; (B) Simulated electric energy density distributions for microwave filter unit cell at 10.5 GHz when *l* = 2.0 mm; (C) Simulated electric energy density distribution for metallic aperture unit cell at 11.82 GHz; (D) Simulated electric energy density distributions for microwave filter unit cell at 11.82 GHz when *l* = 2.0 mm.

Fig 4A shows the electric field energy density distribution in the *xy*-plane for metallic aperture at 10.5 GHz. The incident wave propagates normally onto the metallic plate from the left. There is no electric field energy in the right area, which indicates the incoming microwave cannot transmit through the metallic aperture. When *l* = 2.0 mm, Fig 4B shows the simulated electric field energy density distribution in the *xy*-plane for metallic aperture filled with dielectric particle at 10.5 GHz. Electric field energy does not appear in the right area, which indicates the incoming waves cannot transmit through the metallic aperture filled with dielectric particle when the incident electromagnetic waves fail to correspond to the Mie resonance. Fig 4C shows the electric energy density distribution in the *xy*-plane for metallic aperture at 11.82 GHz. We can see that the microwave still cannot transmit the metallic aperture. As shown in Fig 4D, the electric field energy density distribution is simulated in the *xy*-plane for metallic aperture filled with dielectric particle at 11.82 GHz corresponded to the Mie resonance frequency. At Mie resonance frequency, the dielectric particle acts as a waveguide to efficiently transmit electromagnetic energy through the metallic aperture. The electric energy density depends strongly on the Mie resonance of the dielectric particle, which is in good agreement with that predicted by Eqs (1)–(3). Therefore, the microwave transmits through the metallic aperture at the certain frequency, which can be used to design bandpass filters.

**Fig 5:**
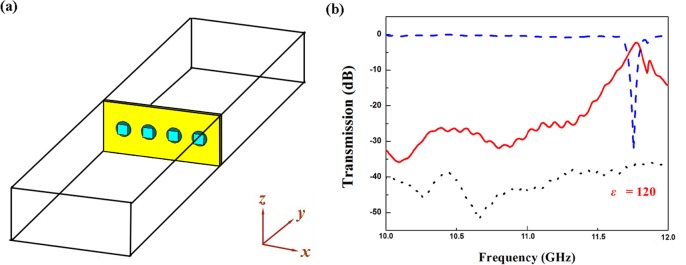
Schematic diagram of the experimental setup and measured transmission apectra for the microwave bandpass filter structure. (A) Schematic diagram of the experimental setup; (B) Measured transmission spectra for the microwave bandpass filter structure when *ε* = 120 (red lines), metallic apertures (black lines) and dielectric particles array without metallic plate (blue lines), respectively.

The schematic diagram experimental setup is shown in Fig 5A. The measured transmission spectra for the microwave bandpass filter structure with *l* = 2.0mm is shown as solid line in Fig 5B. The transmission spectra marked in red has a passband with -10 dB bandwidth of 267 MHz and insertion loss of 2 dB appears at 11.82 GHz, which is induced by Mie resonance of dielectric particles. It can be seen that the experimental results are in good agreement with simulated results. For comparison, transmission spectra for metallic apertures and dielectric particles array without metallic plate are also depicted as black dotted lines and blue dash lines in Fig 5B, respectively. The values of transmission coefficients for metallic apertures is very low (about -40dB), which indicates that the microwave cannot propagate through the structure. A transmission dip appears in the transmission spectrum for dielectric particles array without metallic plate at frequency of 11.82 GHz (Figure E in S1 File), which is corresponding to the Mie resonance.

## Conclusion

A microwave passband filter has been realized based on Mie-resonance extraordinary transmission. The microwave cannot transmit through the metallic apertures for the radius of the aperture is much smaller than the interacted wavelength. By filling the metallic apertures with dielectric particles, an obvious transmission passband with an insertion loss of 2 dB appears. The simulated results show that the center frequency can be influenced by the size and the permittivity of the dielectric particles. The work provides a way to fabricate the microwave bandpass filter.

## Supporting Information

S1 File Data file of the microwave bandpass filter. Figure A. Simulated transmission data file of metallic aperture unit cell. Figure B. Simulated transmission data file of single dielectric particle. Figure C. Simulated transmission data file for the unit cell with a series of length *l* Figure D. Simulated transmission data file for the unit cell with a series of permittivity *ε* Figure E. Measured transmission data file.
(ZIP)
